# Supplementary material for: The Evolution and Characterization of the RNA Interference Pathways in Lophotrochozoa
Source: Genome Biol Evol. 2024 May 7;16(5):evae098. doi: 10.1093/gbe/evae098 (PMC11114477; doi:10.1093/gbe/evae098)
Supplement: evae098_Supplementary_Data [file evae098_supplementary_data.zip › Supplementary_materials.zip!/selected_miRNAs/NoGe_selected_miRNAs/mirdeep_pdf_files/NMRB01000076.1_Notospermus_geniculatus_strain_Ushimado_scaffold76,_whole_genome_shotgun_sequence_2803.pdf]

Provisional ID	:	NMRB01000076.1_Notospermus_geniculatus_strain_Ushimado_scaffold76,_whole_genome_shotgun_sequence_2803
Score total	:	603.5
Score for star read(s)	:	3.9
Score for read counts	:	598.7
Score for mfe	:	1.5
Score for randfold	:	0
Score for cons. seed	:	-0.6
Total read count	:	1186
Mature read count	:	1171
Loop read count	:	0
Star read count	:	15

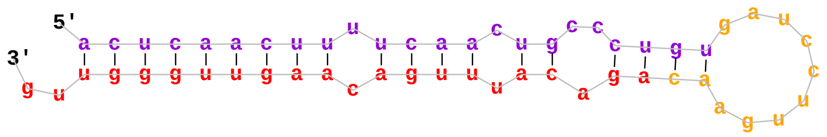

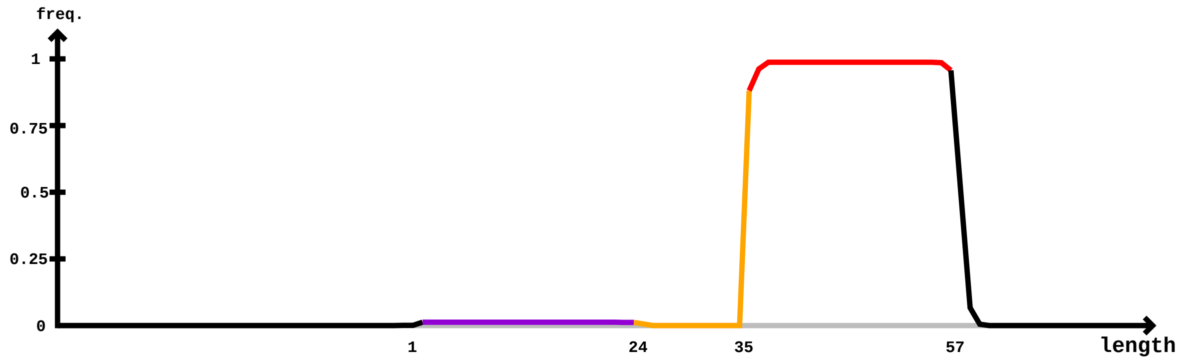

Star Mature

```
5'- caacucaagagaaagugacuggaguugguaauaucgacucaacuuuucaacugcccugugauccuugaacagacauuugacaaguuggguugauguucacaaaccaggcauc   -3'   obs
    caacucaagagaaagugacuggaguugguaauaucgacucaacuuuucaacugcccugugauccuugaacagacauuugacaaguuggguugauguucacaaaccaggcauc         exp
    ..............(((.((((..(((..((((((((((((((((.((((.((..((((.........)))).)).)))).))))))))))))))))..))).)))).))).   reads   mm   sample
    ..................................cgacucaacuuuucaacugcccG.......................................................   1       1    Mm2
    ....................................acucaacuuuucaacugcccugu.....................................................   9       0    Mm2
    ......................................................................agacauuugacaaguugggu......................   1       0    Mm2
    ......................................................................agacauuugacaaguuggguu.....................   6       0    Mm2
    ......................................................................agacauuugCcaaguuggguug....................   1       1    Mm2
    ......................................................................agacauuugacaaguuUgguug....................   1       1    Mm2
    ......................................................................agacauuugaUaaguuggguug....................   2       1    Mm2
    ......................................................................agacauuugacaaguCggguug....................   1       1    Mm2
    ......................................................................agacauuugacaaguuggguug....................   62      0    Mm2
    ......................................................................agacauuugacaaguugAguug....................   1       1    Mm2
    ......................................................................agacauuugacaaguuggguuU....................   1       1    Mm2
    ......................................................................agacauuugacaaguuggguugU...................   170     1    Mm2
    ......................................................................agacauuugacaaguuggguugG...................   2       1    Mm2
    ......................................................................agacauuugacaaguuggguuga...................   22      0    Mm2
    ......................................................................agacauuugacaaguuggguugUu..................   6       1    Mm2
    ......................................................................agacauuugacaaguuggguugaG..................   1       1    Mm2
    ......................................................................agacauuugacaaguuggguugau..................   2       0    Mm2
    .......................................................................gacauuugacaaguuggguuga...................   13      0    Mm2
    ........................................................................acauuugacaaguuggguugU...................   1       1    Mm2
    ........................................................................acauuugacaaguuggguuga...................   1       0    Mm2
    ........................................................................acauuugacaaguuggguugau..................   6       0    Mm2
    ........................................................................acauuugacaaguuggguugauU.................   1       1    Mm2

    ......................................................................agacauuugacaaguugggu......................   1       0    Mm1
    ......................................................................agacauuugacaaguuggguug....................   14      0    Mm1
    ......................................................................agacauuugacaaguuggguugU...................   24      1    Mm1
    ......................................................................agacauuugacaaguuggguugUu..................   3       1    Mm1

    ......................................................................agacauuugacaaguuCgguug....................   1       1    Fm1
    ......................................................................agacauuugacaaguugCguug....................   1       1    Fm1
    ......................................................................agacauuugacaaguuggguCg....................   1       1    Fm1
    ......................................................................agacauuugacaaguuggguGg....................   2       1    Fm1
    ......................................................................agacauuugacaaguuggguug....................   62      0    Fm1
```

```
                                   Star                             Mature
caacucaagagaaagugacuggaguugguaauaucgacucaacuuuucaacugcccugugauccuugaacagacauuugacaaguuggguugauguucacaaaccaggcauc
......................................................................aAacauuugacaaguuggguug....................    1     1    Fm1
......................................................................agacauuugacaaguuggguuga...................    11    0    Fm1
......................................................................agacauuugacaaguuggguugU...................    70    1    Fm1
......................................................................agacauuugacaaguuggguugG...................    1     1    Fm1
......................................................................agacauuugacaaguuggguugUu..................    4     1    Fm1
......................................................................agacauuugacaaguuggguugau..................    3     0    Fm1
......................................................................agacauuugacaaguuggguugaA.................    2     1    Fm1
.......................................................................gacauuugacaaguuggguuga...................    8     0    Fm1
........................................................................acauuugacaaguugggCugau..................    1     1    Fm1
........................................................................acauuugacaaguuggguugau..................    4     0    Fm1
........................................................................acauuugacaaguuggguugauA.................    5     1    Fm1

......................................................................agacauuugacaaUuuggguu.....................    1     1    Fm2
......................................................................agacauuugacaaguuggguu.....................    14    0    Fm2
......................................................................agacauuugacaaguuggguGg....................    1     1    Fm2
......................................................................agacauuugacaaguuggguug....................    180   0    Fm2
......................................................................agacauuugacaaguugAguug....................    2     1    Fm2
......................................................................agacauuugacaGguuggguug....................    4     1    Fm2
......................................................................agacauuugacaaguugCguug....................    3     1    Fm2
......................................................................agacGuuugacaaguuggguuga...................    1     1    Fm2
......................................................................agacauuugacaaguuggguugU...................    94    1    Fm2
......................................................................agacauuugacaaguuggguugG...................    2     1    Fm2
......................................................................agacauuugacaaguuggguuga...................    50    0    Fm2
......................................................................agacauuugacaGguuggguuga...................    4     1    Fm2
......................................................................agacauuugacaaguuggguugUu..................    8     1    Fm2
......................................................................agacauuugacaaguuggguugau..................    5     0    Fm2
.......................................................................gacauuugacaaguuggguug....................    6     0    Fm2
.......................................................................gacauuugacaaguuggguuga...................    41    0    Fm2
.......................................................................gacauuugacaaguuggAuuga...................    1     1    Fm2
........................................................................acauuugacaaguuggguugau..................    9     0    Fm2
........................................................................acauuugacaaguuggguugaA..................    2     1    Fm2

....................................acucaacuuuucaacugcccugu.....................................................    4     0    Mm3
......................................................................agacauuugacaaguuggguu.....................    2     0    Mm3
......................................................................agacauuugacaaguuggguuU....................    1     1    Mm3
......................................................................agacauuugacaaguuggguuC....................    1     1    Mm3
......................................................................agacauuugacaaguuggguug....................    10    0    Mm3
......................................................................agacauuugacaaguuggguugU...................    45    1    Mm3
......................................................................agacauuugacaaguuggguuga...................    3     0    Mm3
......................................................................agacauuugacaaguuggguugUu..................    3     1    Mm3
......................................................................agacauuugacaaguuggguugau..................    2     0    Mm3
......................................................................agacauuugacaaguuggguugaA.................    1     1    Mm3

....................................acucaacuuuucaacugcccugu.....................................................    1     0    Fm3
......................................................................agacauuugacaaguuggguu.....................    10    0    Fm3
......................................................................agacauuugacaaguugUguu.....................    1     1    Fm3
......................................................................agacauuugacaaguuggguug....................    54    0    Fm3
......................................................................agacauuugacaaguuggguuU....................    1     1    Fm3
......................................................................agacauuugacaaguuggAuuga...................    1     1    Fm3
......................................................................agacauuugacaaguuggguuga...................    18    0    Fm3
......................................................................agacauuugacaaguuggguugU...................    41    1    Fm3
......................................................................agacauuugacaaguuggguugau..................    2     0    Fm3
.......................................................................gacauuugacaaguuggguug....................    2     0    Fm3
.......................................................................gacauuugacaaguuggguugU...................    2     1    Fm3
.......................................................................gacauuugacaaguuggguuga...................    14    0    Fm3
.......................................................................gacauuugacaaguuggguugaA..................    6     1    Fm3
.......................................................................gacauuugacaaguuggguugau..................    3     0    Fm3
```
